# Willingness and uptake of the COVID-19 testing and vaccination in urban China during the low-risk period: a cross-sectional study

**DOI:** 10.1186/s12889-022-12969-5

**Published:** 2022-03-21

**Authors:** Suhang Song, Shujie Zang, Liubing Gong, Cuilin Xu, Leesa Lin, Mark R. Francis, Zhiyuan Hou

**Affiliations:** 1 Taub Institute for Research in Alzheimer’s Disease and the Aging Brain, Columbia University, New York, NY USA; 2 School of Public Health, Fudan University, Shanghai, China; 3 Chizhou Center for Disease Prevention and Control, Chizhou, Anhui province China; 4 Yuhuatai Center for Disease Prevention and Control, Nanjing, China; 5 Department of Infectious Disease Epidemiology, London School of Hygiene & Tropical Medicine, London, UK; 6 Laboratory of Data Discovery for Health, Hong Kong Science Park, Hong Kong SAR, China; 7 Health Sciences Unit, Faculty of Social Sciences, Tampere University, Tampere, Finland; 8 National Health Commission Key Laboratory of Health Technology Assessment, Fudan University, Shanghai, China

**Keywords:** Willingness, Uptake, COVID-19 testing, COVID-19 vaccination

## Abstract

**Background:**

Regular testing and vaccination are effective measures to mitigate the ongoing COVID-19 pandemic. Evidence on the willingness and uptake of the COVID-19 testing is scarce, and the willingness and uptake of vaccination may change as the pandemic evolves. This study aims to examine willingness and uptake of COVID-19 testing and vaccination during a low-risk period of the COVID-19 pandemic in urban China.

**Methods:**

A cross-sectional online survey was conducted among 2244 adults in urban China. Descriptive analyses were performed to compare the respondents’ willingness and uptake of COVID-19 testing and vaccination. Multivariate logistic regressions were fitted to investigate factors associated with the willingness and uptake of the two measures.

**Results:**

In early 2021, about half (52.45%) of the respondents had received or scheduled a COVID-19 test at least once, and a majority (95.63%) of the respondents were willing to receive testing. About two-thirds (63.28%) of the respondents had received/scheduled or were willing to receive a COVID-19 vaccine. Willingness and uptake of COVID-19 testing were not associated with socio-demographic characteristics, except for occupation. Being of older age, migrants, having higher educational attainment and secure employment were associated with a higher uptake of COVID-19 vaccination among the surveyed respondents, while willingness to vaccinate was consistent across socio-demographic characteristics among those who had not been vaccinated.

**Conclusions:**

By early 2021, Chinese adults expressed almost universal willingness of COVID-19 testing and over half of adults have been tested, while the willingness and uptake of COVID-19 vaccination were relatively low at the low-risk period of the COVID-19 pandemic. Maintaining willingness of COVID-19 vaccination is critical and necessary, especially when the pandemic evolved into a low-risk period.

## Introduction

Testing and vaccination are two effective measures to mitigate and prevent the transmission of COVID-19 [1–4]. Testing can be used to diagnose COVID-19 by detecting both symptomatic and asymptomatic patients, and can also trace confirmed cases and their close contacts, especially when outbreaks surge [3, 5]. The vaccine is expected to play an important role in preventing serious complications from SARS-CoV-2 infections and establishing herd immunity to protect populations from COVID-19 infections [6]. The vaccinations of COVID-19 are being promoted and scaled up globally; in China, the government enacted the emergency use authorization of COVID-19 vaccines in June 2020 [7, 8], and subsequently approved COVID-19 vaccines for general use in December 2020 [8, 9]. By the end of March 2020, the peak of the pandemic has passed in China, and the number of new confirmed cases per day rapidly declined to less than 10; most of these cases were imported from abroad [4, 10, 11]. With COVID-19 resurging in some regions, mass COVID-19 testing and vaccination strategies have been adopted to track and control sporadic outbreaks in many cities such as Beijing [12] and Qingdao [13]. Understanding the willingness to receive and uptake of testing and vaccinations can help design and implement policies to improve access to and acceptance of COVID-19 tests and vaccination, which is important for effectively promoting pandemic mitigation and prevention strategies.

Previous reviews and empirical studies have investigated the public’s willingness and uptake of COVID-19 vaccinations during the high-risk period of COVID-19 pandemic, however, it is unknown on the willingness of COVID-19 vaccination when the pandemic evolved into the low-risk period [14–26]. These studies reported that the willingness to be vaccinated varied by geographic area [27–34], socio-demographic characteristics such as age and occupation [29–31, 35–41], and COVID-19 disease and vaccine risk perceptions [29, 33, 42–47]. A previous survey from China estimated a high willingness to be vaccinated against COVID-19 at the beginning of the pandemic, which declined as the pandemic became normalized due to the reduced perception of COVID-19 risk among the public [48]. Therefore, it is crucial to continue assessing the public’s willingness to be vaccinated as their attitudes and risk perceptions may change over time. Tracking the willingness and uptake of COVID-19 vaccination helps understand the progress of herd immunity and determine how the willingness changes over time, and may offer support in improving the COVID-19 vaccination policies. Health disparities [49, 50], especially in vaccination uptake also need to be further studied, as only a few studies to date have examined the uptake of the COVID-19 vaccination by population characteristics. In addition, the tests of COVID-19 serve as an important complementary measure to prevent and control spikes in SARS-CoV-2 cases, enabling disease diagnosis and tracing the confirmed cases [51–56]. Nearly 160 million tests have been performed in China, as of August 6^th^, 2021 [57]. However, studies on the willingness to be tested and actual uptake of COVID-19 tests are scarce; updated estimates of COVID-19 testing can help identify the population subgroups to be targeted by health education interventions in China.

Therefore, we conducted a cross-sectional survey in early 2021, when COVID-19 testing was being used as a primary measure to detect the sporadic outbreaks of SARS-CoV-2 cases [48] and after the COVID-19 vaccine had been officially approved for use among the general public [8]. This study aims to examine the willingness and uptake of COVID-19 testing and vaccination during the low-risk period of the COVID-19 pandemic in China.

## Methods

### Study Design, Population and Sampling

We conducted an anonymous online cross-sectional survey to collect information on the willingness and uptake of the COVID-19 testing and vaccination and their associated factors among adults aged 18 years and older in two cities (Nanjing and Chizhou) from January 29 to February 4, 2021. Nanjing city in eastern Jiangsu province and Chizhou city in central Anhui province had a gross domestic product (GDP) per capita of CNY 165,681 among 7.10 million population and of CNY 56,217 among 1.62 million population, [58] respectively, making them good representatives of urban China [59]. This study employed snowball sampling to enroll the study participants from four to eight community health centers in each city and from the local Centers for Disease Control and Prevention (CDC). Participants could access the questionnaire through a social media platform, WeChat, which has 1.1 billion active users. Those who completed the survey were encouraged to share a link of the questionnaire and invite their colleagues or friends to participate. To avoid repeated participations, each WeChat account was permitted to fill out the questionnaire only once, and only devices having Internet Protocol addresses were able to submit their responses successfully. It took about 3–5 min to complete the self-administered questionnaire and the respondents were given a gift worth roughly CNY 5 after they completed the survey.

A total of 2250 respondents answered the questionnaire independently and provided e-consent for their participation in the survey. Six incomplete questionnaires or questionnaires completed under two minutes were excluded from the analysis, and a total of 2244 respondents were included in the study. The study was approved by the Institutional Review Board of the School of Public Health, Fudan University (IRB#2020–12-0861).

### Measures

The self-administered questionnaire was designed based on previous literature and pre-tested among ten respondents, who were excluded from the analysis. The questionnaire included questions on the respondent’s socio-demographic characteristics, self-reported health status, awareness of the COVID-19 pandemic, perceived susceptibility and severity of the COVID-19, and willingness to receive and uptake of COVID-19 testing and vaccination.

The outcomes of interest for this study were the willingness and uptake of COVID-19 testing and vaccination. Uptake of the COVID-19 testing was measured with the question “Have you ever received a COVID-19 test before?” Response options included “already tested,” “scheduled,” and “haven't been tested or scheduled.” Respondents who responded “already tested” or “scheduled” were classified into the uptake group, and were further asked their reasons for receiving testing, including community-wide mass testing led by governments, mandatory testing policies for travel, and personal health needs. Respondents who responded “haven't been tested or scheduled” were included in the group who haven’t received  the test. The willingness to receive the COVID-19 testing was enquired of all participants with a five-point Likert scale question, with options including “willing,” ‘‘somewhat willing,” ‘‘undecided,” ‘‘somewhat not willing,” and ‘‘not willing.” Responses of “willing” and ‘‘somewhat willing” were classified as “willing”, while those who chose the other three options were assigned as “unwilling”. Similar to the uptake of COVID-19 testing, uptake of the COVID-19 vaccination was assessed by whether the participants had been vaccinated against COVID-19; those who chose either “vaccinated” or “scheduled” were added to the “vaccinated” group. Only respondents who had not been vaccinated or not scheduled a COVID-19 vaccination were asked for their willingness to be vaccinated in the future. We assigned participants who had not received or scheduled a vaccination to report their willingness to be vaccinated, as COVID-19 vaccination needs to be received only one time under the policy during the survey time, while COVID-19 tests need to be received multiple times as needed. However, willingness to continue to be tested still matters for those who had already received a COVID-19 test, and thus needed to be analyzed.

The socio-demographic characteristics collected included location, residency, age, gender, marital status, educational attainment, occupation, and annual income. Location, residency and gender were dichotomized into Nanjing city versus Chizhou city, local residents versus migrants, and male versus female, respectively. Participant’s age was categorized into four groups, including 18–25, 26–35, 36–45, ≥ 46 years old. Marital status was classified into single, married and divorced/widow. Educational attainment was grouped into middle school or lower, high school or technical secondary school, junior college, and bachelor’s degree or higher. Occupation was categorized into four groups: government agency, service industry, manufacturing industry or agriculture, and others. Annual individual income was grouped into < 20,000, 20,000–50,000, 50,000–100,000, 100,000–200,000 and > 200,000 Chinese Yuan (CNY). Self-reported health status was assessed on a five-point Likert scale ranging from very good (1) to very poor (5) and dichotomized into “good” (very good and good) versus “poor” (fair, poor and very poor). Awareness of and perceived susceptibility to COVID-19 were also assessed on a five-point Likert scale—very high, high, not sure, low, and very low. Respondents who selected the first two options were classified into the “high” group, and those who selected the other three options were assigned to the “low” group. We also asked participants to answer a question on how severe they considered COVID-19 infections to be, “How do you think your symptoms would be if you were infected with COVID-19?”, with the following options—severe, moderate, mild, asymptomatic and unsure. These responses were dichotomized into “severe or moderate” versus “mild” (including asymptomatic and unsure).

### Statistical analysis

Descriptive analyses were performed to compare the characteristics of respondents by their willingness and uptake of COVID-19 testing and vaccination. Since all the assessed factors were categorical variables, chi-square tests were used to compare participant’ characteristics across the willingness and uptake groups. Multivariate logistic regression models were fitted to investigate factors associated with the willingness and uptake of COVID-19 testing and vaccination separately, with the adjusted odds ratio (aOR) and 95% confidence intervals (CIs) being calculated. Two-sided *P* < 0.05 indicated significance.

## Results

Table 1 presents a descriptive summary of the characteristics of the survey respondents. Respondents were more likely to be female (68.81%, 1544/2244), aged 26–35 years (46.48%, 1043/2244), local residents (86.68%, 1945/2244), married (85.16%, 1911/2244), have a bachelor’s degree or higher (42.02%, 943/2244), working in a government agency (34.49%, 774/2244), have an annual income of 50,000–100,000 Chinese Yuan (31.24%, 701/2244), and report being in good health (90.24%, 2025/2244). In addition, the majority of respondents portrayed low perceived susceptibility (93.49%, 2098/2244) and mild perceived severity of COVID-19 (81.11%, 1820/2244) although they had high awareness of the COVID-19 (92.78%, 2082/2244). Respondents were almost equally distributed between the two selected cities.

**Table 1:** Characteristics of study respondents by the willingness and uptake of COVID-19 testing

Characteristics	Total (%)	Uptake of COVID-19 testing		Willingness of COVID-19 testing	
		Tested/scheduled (%)	Not tested/scheduled (%)	Willing (%)	Unwilling (%)
Total	2244 (100)	1177 (52.45)	1067 (47.55)	2146 (95.63)	98 (4.37)
City		***P* < 0.001**		***P* < 0.001**	
Nanjing	1091 (48.62)	626 (57.38)	465 (42.62)	1024 (93.86)	67 (6.14)
Chizhou	1153 (51.38)	551 (47.79)	602 (52.21)	1122 (97.31)	31 (2.69)
Gender		*P* = 0.638		*P* = 0.152	
Male	700 (31.19)	362 (51.71)	338 (48.29)	663 (94.71)	37 (5.29)
Female	1544 (68.81)	815 (52.78)	729 (47.22)	1483 (96.05)	61 (3.95)
Age (years)		*P* = 0.099		*P* = 0.199	
18–25	218 (9.71)	119 (54.59)	99 (45.41)	208 (95.41)	10 (4.59)
26–35	1043 (46.48)	519 (49.76)	524 (50.24)	998 (95.69)	45 (4.31)
36–45	567 (25.27)	317 (55.91)	250 (44.09)	549 (96.83)	18 (3.17)
> = 46	416 (18.54)	222 (53.37)	194 (46.63)	391 (93.99)	25 (6.01)
Residency		*P* = 0.309		***P* = 0.003**	
Local residents	1945 (86.68)	1012 (52.03)	933 (47.97)	1870 (96.14)	75 (3.86)
Migrants	299 (13.32)	165 (55.18)	134 (44.82)	276 (92.31)	23 (7.69)
Marital status		***P* = 0.044**		*P* = 0.352	
Single	283 (12.61)	168 (59.36)	115 (40.64)	266 (93.99)	17 (6.01)
Married	1911 (85.16)	984 (51.49)	927 (48.51)	1832 (95.87)	79 (4.13)
Divorced/widow	50 (2.23)	25 (50)	25 (50)	48 (96)	2 (4)
Educational attainment		***P* < 0.001**		*P* = 0.343	
Middle school or lower	341 (15.20)	131 (38.42)	210 (61.58)	329 (96.48)	12 (3.52)
High school or technical secondary school	402 (17.91)	163 (40.55)	239 (59.45)	378 (94.03)	24 (5.97)
Junior college	558 (24.87)	289 (51.79)	269 (48.21)	536 (96.06)	22 (3.94)
Bachelor's degree or higher	943 (42.02)	594 (62.99)	349 (37.01)	903 (95.76)	40 (4.24)
Occupation		***P* < 0.001**		***P* = 0.001**	
Government agency	774 (34.49)	582 (75.19)	192 (24.81)	758 (97.93)	16 (2.07)
Service industry	580 (25.85)	273 (47.07)	307 (52.93)	547 (94.31)	33 (5.69)
Manufacturing industry or agriculture	302 (13.46)	102 (33.77)	200 (66.23)	290 (96.03)	12 (3.97)
Others	588 (26.20)	220 (37.41)	368 (62.59)	551 (93.71)	37 (6.29)
Annual individual income		***P* = 0.001**		*P* = 0.821	
< 20 k	244 (10.87)	107 (43.85)	137 (56.15)	230 (94.26)	14 (5.74)
20-50 k	373 (16.62)	181 (48.53)	192 (51.47)	359 (96.25)	14 (3.75)
50-100 k	701 (31.24)	359 (51.21)	342 (48.79)	672 (95.86)	29 (4.14)
100-200 k	606 (27.01)	339 (55.94)	267 (44.06)	579 (95.54)	27 (4.46)
> 200 k	320 (14.26)	191 (59.69)	129 (40.31)	306 (95.63)	14 (4.38)
Self-reported health status		*P* = 0.382		*P* = 0.232	
Good	2025 (90.24)	1056 (52.15)	969 (47.85)	1940 (95.80)	85 (4.20)
Poor	219 (9.76)	121 (55.25)	98 (44.75)	206 (94.06)	13 (5.94)
Awareness of COVID-19		***P* = 0.014**		***P* < 0.001**	
High	2082 (92.78)	1107 (53.17)	975 (46.83)	2007 (96.40)	75 (3.60)
Low	162 (7.22)	70 (43.21)	92 (56.79)	139 (85.80)	23 (14.20)
Perceived susceptibility of COVID-19		***P* < 0.001**		*P* = 0.157	
High	146 (6.51)	116 (79.45)	30 (20.55)	143 (97.95)	3 (2.05)
Low	2098 (93.49)	1061 (50.57)	1037 (49.43)	2003 (95.47)	95 (4.53)
Perceived severity of COVID-19		***P* = 0.034**		*P* = 0.696	
Severe or moderate	424 (18.89)	242 (57.08)	182 (42.92)	404 (95.28)	20 (4.72)
Mild	1820 (81.11)	935 (51.37)	885 (48.63)	1742 (95.71)	78 (4.29)

**Fig. 1:**
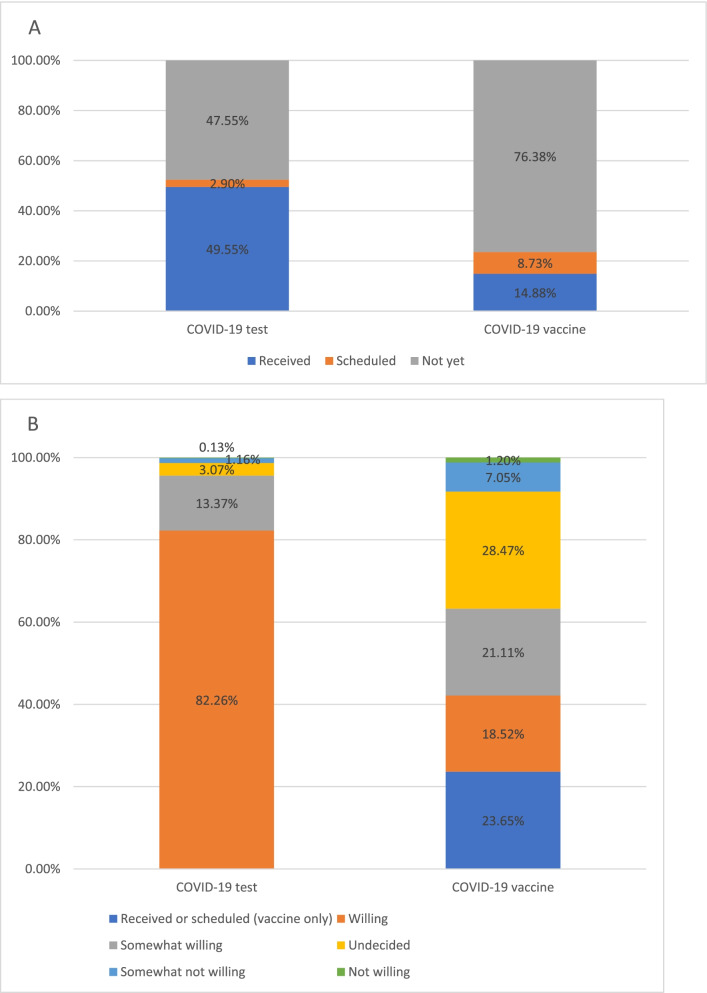
Willingness and uptake of COVID-19 testing and vaccination. **A** Uptake of COVID-19 testing and vaccination. **B** Willingness of COVID-19 testing and vaccination. Note: Willingness of COVID-19 vaccination included 3 missing values

**Fig. 2:**
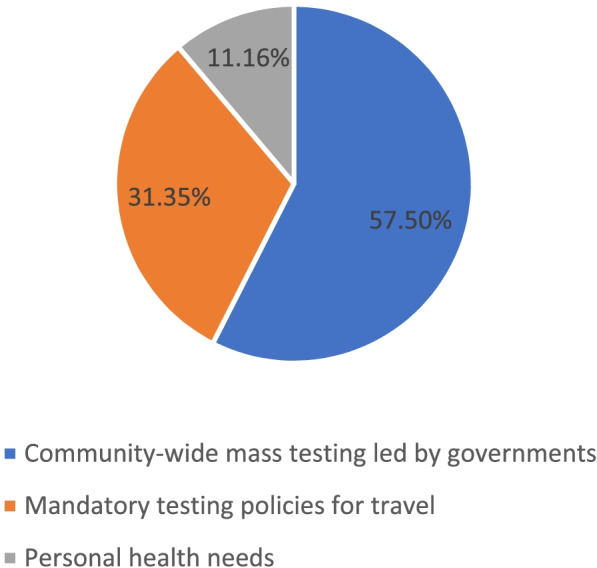
Reasons for the uptake of COVID-19 testing. Note: Three participants who have received the COVID-19 test didn't report their reasons of receiving the test, so the sample size for the testing reason question was limited to 1174

Figure 1 presents the survey respondent’s willingness and uptake of COVID-19 testing and vaccination. At the time of the survey, 52.45% (1177/2244) and 23.62% (530/2244) of respondents had received or scheduled at least one COVID-19 test and COVID-19 vaccine, respectively (Fig. 1A). Among the respondents who had ever received or scheduled a COVID-19 test, more than half (57.50% [675/1174]) did so because of community-wide mass testing led by governments, followed by mandatory testing policies for travel (31.35%, 368/1174), and personal health needs (11.16%, 131/1174) (Fig. 2). Concerning willingness to receive COVID-19 tests or vaccines, the majority (95.63% [2146/2244]) reported being willing to receive a COVID-19 test, and 63.28% (1418/2241) either received, scheduled, or reported being willing to receive a COVID-19 vaccine (Fig. 1B).

Table 1 also contains a descriptive summary of the respondents’ characteristics stratified by their willingness and uptake of COVID-19 testing. More respondents in Nanjing city had received or scheduled a COVID-19 test at least once (57.38%, 626/1091), compared to respondents in Chizhou city (47.79%, 551/1153). About three-fourths (75.19%, 582/774) of respondents who worked in a government agency had ever received or scheduled a COVID-19 test, than respondents working in service, manufacturing, agriculture, and other industries. Respondents who had completed junior college or received a bachelor’s degree or higher had a greater uptake rate of COVID-19 testing—51.79% (289/558) and 62.99% (594/943), respectively. However, fewer respondents (39.57% [294/743]) with lower educational attainment (high school and lower) had ever received/scheduled a COVID-19 test. More than half of the respondents with an annual individual income over 50,000 Chinese Yuan had received or scheduled a test at least once. A similar proportion (53.17% [1107/2082] of respondents who reported a high level of COVID-19 awareness had ever received or scheduled a COVID-19 test. Across each group, more than 85.80% of respondents were willing to receive a test.

Table 2 presents factors associated with the willingness and uptake of COVID-19 testing using multivariate logistic regressions. Location, occupation, awareness of and perceived susceptibility to COVID-19 were significantly associated with receiving testing. Compared to respondents living in Nanjing city, fewer respondents in Chizhou city had ever received or scheduled a COVID-19 test (aOR = 0.765, 95% CI = 0.619–0.946). However, more respondents in Chizhou city were willing to receive testing than in Nanjing city (aOR = 2.097, 95% CI = 1.248–3.524). Compared to respondents who reported working in a government agency, respondents with less secure occupations (service, manufacturing, agricultural or other industries) had a significantly lower uptake and willingness to receive COVID-19 testing. Associations between the other socio-demographic factors (i.e., marital status, educational attainment, annual individual income) and the willingness to receive and uptake of COVID-19 testing failed to reach statistical significance in the multivariate analysis. The uptake rate among respondents with a high perceived susceptibility to COVID-19 was nearly three times (aOR: 2.719, 95% CI = 1.739–4.251) higher than those with lower perceived susceptibility. The willingness to receive testing among respondents with high COVID-19 awareness was 4.318 times (95% CI = 2.550–7.314) higher than those with low awareness.

**Table 2:** Factors associated with the willingness and uptake of COVID-19 testing

	Uptake of COVID-19 testing (aOR)	95%CI	Willingness of COVID-19 testing (aOR)	95%CI
City (ref: Nanjing)				
Chizhou	0.765*	(0.619—0.946)	2.097**	(1.248—3.524)
Gender (ref: male)				
Female	1.116	(0.912—1.367)	1.436	(0.913—2.259)
Age (ref: 18–25 years)				
26–35	0.703	(0.487—1.013)	0.707	(0.305—1.641)
36–45	0.725	(0.478—1.098)	0.750	(0.272—2.066)
> = 46	0.832	(0.537—1.290)	0.429	(0.155—1.189)
Residency (ref: local residents)				
Migrants	1.179	(0.893—1.555)	0.606	(0.348—1.055)
Marital status (ref: single)				
Married	0.912	(0.652—1.275)	1.229	(0.609—2.479)
Divorced/widow	0.783	(0.396—1.547)	1.511	(0.305—7.494)
Educational attainment (ref: middle school or lower)				
High school or technical secondary school	0.862	(0.630—1.179)	0.552	(0.263—1.161)
Junior college	1.022	(0.747—1.398)	0.717	(0.326—1.576)
Bachelor's degree or higher	1.252	(0.893—1.756)	0.686	(0.302—1.558)
Occupation (ref: government agency)				
Service industry	0.307**	(0.239—0.395)	0.403**	(0.212—0.767)
Manufacturing industry or agriculture	0.190**	(0.138—0.261)	0.570	(0.253—1.284)
Others	0.216**	(0.164—0.283)	0.316**	(0.162—0.617)
Annual individual income (ref: < 20 k)				
20-50 k	1.207	(0.854—1.706)	1.295	(0.591—2.837)
50-100 k	1.021	(0.739—1.410)	1.294	(0.640—2.617)
100-200 k	0.895	(0.632—1.267)	1.230	(0.580—2.609)
> 200 k	0.804	(0.539—1.199)	1.196	(0.500—2.860)
Self-reported health status (ref: good)				
Poor	1.087	(0.795—1.486)	0.833	(0.436—1.594)
Awareness of COVID-19 (ref: low)				
High	1.286	(0.910—1.817)	4.318**	(2.550—7.314)
Perceived susceptibility of COVID-19 (ref: low)				
High	2.719**	(1.739—4.251)	2.261	(0.673—7.597)
Perceived severity of COVID-19 (ref: mild)				
Severe or moderate	1.006	(0.792—1.278)	0.829	(0.486—1.417)

^*^p < 0.05. **p < 0.01

*aOR* adjusted odds ratio

The uptake of COVID-19 vaccination differed across socio-demographic characteristics, awareness of and perceived susceptibility to COVID-19 (Table 3). Among the 2244 respondents, there were significant differences in the uptake of COVID-19 vaccination by location, age, educational attainment, occupation, annual income, and COVID-19 awareness. When participant’s occupations were considered, the percentages of respondents who had not been or scheduled a vaccination ranged from 53.10% (411/774) for those working in government agencies to 92.72% (280/302) for those working in the manufacturing industry or agriculture. Respondents with low perceived susceptibility to COVID-19 were more likely to have not received or scheduled a COVID-19 vaccination (78.60%, 1649/2098), while those with high perceived susceptibility had a higher uptake rate of COVID-19 vaccinations (55.84%, 81/146). Regarding willingness to receive COVID-19 vaccinations among the 1711 respondents who had not been vaccinated or did not have vaccinations scheduled, 49.12% (419/853) from Nanjing city and 54.66% (469/858) from Chizhou city reported being willing to receive the vaccination. A large proportion of respondents in both the high and low perceived susceptibility to COVID-19 categories were willing to receive a vaccination, accounting for 65.63% (42/64) and 51.37% (846/1647), respectively. Respondents with high COVID-19 awareness were more willing to receive COVID-19 vaccination (54.20%, 851/1570), while those with low awareness had a lower willingness to receive COVID-19 vaccination (26.24%, 37/141).

**Table 3:** Characteristics of study respondents by the willingness and uptake of COVID-19 vaccination

Characteristics	Uptake of COVID-19 vaccination		Willingness of COVID-19 vaccination among those not vaccinated	
	Vaccinated /scheduled (%)	Not vaccinated/scheduled (%)	Willing (%)	Unwilling (%)
Total	530 (23.62)	1714 (76.38)	888 (51.90)	823 (48.10)
City	***P* = 0.040**		***P* = 0.022**	
Nanjing	237 (21.72)	854 (78.28)	419 (49.12)	434 (50.88)
Chizhou	293 (25.41)	860 (74.59)	469 (54.66)	389 (45.34)
Gender	*P* = 0.252		*P* = 0.082	
Male	176 (25.14)	524 (74.86)	288 (55.07)	235 (44.93)
Female	354 (22.93)	1190 (77.07)	600 (50.51)	588 (49.49)
Age (years)	***P* < 0.001**		*P* = 0.799	
18–25	46 (21.10)	172 (78.90)	90 (52.33)	82 (47.67)
26–35	183 (17.55)	860 (82.45)	454 (52.98)	403 (47.02)
36–45	186 (32.80)	381 (67.20)	193 (50.66)	188 (49.34)
> = 46	115 (27.64)	301 (72.36)	151 (50.17)	150 (49.83)
Residency	*P* = 0.499		*P* = 0.822	
Local residents	464 (23.86)	1481 (76.14)	766 (51.79)	713 (48.21)
Migrants	66 (22.07)	233 (77.93)	122 (52.59)	110 (47.41)
Marital status	*P* = 0.667		*P* = 0.277	
Single	70 (24.73)	213 (75.27)	113 (53.05)	100 (46.95)
Married	446 (23.34)	1465 (76.66)	761 (52.05)	701 (47.95)
Divorced/widow	14 (28)	36 (72)	14 (38.89)	22 (61.11)
Educational attainment	***P* < 0.001**		*P* = 0.784	
Middle school or lower	33 (9.68)	308 (90.32)	155 (50.49)	152 (49.51)
High school or technical secondary school	53 (13.18)	349 (86.82)	188 (54.18)	159 (45.82)
Junior college	149 (26.70)	409 (73.30)	209 (51.10)	200 (48.90)
Bachelor's degree or higher	295 (31.28)	648 (68.72)	336 (51.85)	312 (48.15)
Occupation	***P* < 0.001**		*P* = 0.321	
Government agency	363 (46.90)	411 (53.10)	221 (53.77)	190 (46.23)
Service industry	101 (17.41)	479 (82.59)	251 (52.51)	227 (47.49)
Manufacturing industry or agriculture	22 (7.28)	280 (92.72)	152 (54.29)	128 (45.71)
Others	44 (7.48)	544 (92.52)	264 (48.71)	278 (51.29)
Annual individual income	***P* = 0.007**		*P* = 0.973	
< 20 k	37 (15.16)	207 (84.84)	107 (52.20)	98 (47.80)
20-50 k	81 (21.72)	292 (78.28)	151 (51.71)	141 (48.29)
50-100 k	173 (24.68)	528 (75.32)	270 (51.14)	258 (48.86)
100-200 k	151 (24.92)	455 (75.08)	235 (51.76)	219 (48.24)
> 200 k	88 (27.50)	232 (72.50)	125 (53.88)	107 (46.12)
Self-reported health status	*P* = 0.072		***P* = 0.050**	
Good	489 (24.15)	1536 (75.85)	808 (52.71)	725 (47.29)
Poor	41 (18.72)	178 (81.28)	80 (44.94)	98 (55.06)
Awareness of COVID-19	***P* < 0.001**		***P* < 0.001**	
High	510 (24.50)	1572 (75.50)	851 (54.20)	719 (45.80)
Low	20 (12.35)	142 (87.65)	37 (26.24)	104 (73.76)
Perceived susceptibility of COVID-19	***P* < 0.001**		***P* = 0.025**	
High	81 (55.48)	65 (44.52)	42 (65.63)	22 (34.38)
Low	449 (21.40)	1649 (78.60)	846 (51.37)	801 (48.63)
Perceived severity of COVID-19	*P* = 0.103		*P* = 0.163	
Severe or moderate	113 (26.65)	311 (73.35)	172 (55.48)	138 (44.52)
Mild	417 (22.91)	1403 (77.09)	716 (51.11)	685 (48.89)

Only 1714 participants who haven't received or scheduled a COVID-19 vaccine were asked their willingness. Among them, three participants didn't report their willingness, so the sample size for the vaccination willingness question was limited to 1711

Table 4 presents factors associated with the willingness and uptake of COVID-19 vaccinations in the study sites. Location, age, residence, educational attainment, occupation, self-reported health status, and perceived susceptibility to COVID-19 were significantly associated with uptake of COVID-19 vaccination among the survey respondents. Respondents living in Chizhou city, compared to Nanjing city, had a higher uptake rate of COVID-19 vaccination (aOR = 1.928, 95% CI = 1.488–2.498). Persons aged 46 years or older (aOR = 2.012, 95% CI = 1.133–3.574), compared to those aged 18–25 years, were more likely to receive or schedule a vaccination, while respondents who had poor perceived health were less likely to receive or schedule a vaccination (aOR = 0.540, 95% CI = 0.352–0.829). Vaccination uptake among migrants was 1.479 times (95% CI = 1.040–2.104) higher than among local residents. Respondents who had educational attainment of junior college or higher and worked in government agencies had higher uptake of COVID-19 vaccinations than those with lower educational attainment or less secure occupations (i.e., industry). The vaccination uptake rate among respondents with high perceived susceptibility to COVID-19 was 3.457 times (95% CI = 2.298–5.199) higher than those with low perceived susceptibility. In terms of willingness to receive COVID-19 vaccination, among 1711 respondents who had not been or scheduled a vaccination, more respondents in Chizhou city reported being willing to receive a COVID-19 vaccination than those living in Nanjing city (aOR = 1.404, 95% CI = 1.110–1.776). Willingness to be vaccinated among respondents with high awareness of and perceived susceptibility to COVID-19 was 3.391 (95% CI = 2.285–5.032) and 1.950 (95% CI = 1.119–3.398) times higher than those with low awareness and perceived susceptibility, respectively. Other socio-demographic characteristics were not associated with the willingness to receive a COVID-19 vaccination among those who had been vaccinated.

**Table 4:** Factors associated with the willingness and uptake of COVID-19 vaccination

	Uptake of COVID-19 vaccination (aOR)	95%CI	Willingness of COVID-19 vaccination (aOR)	95%CI
City (ref:Nanjing)				
Chizhou	1.928**	(1.488—2.498)	1.404**	(1.110—1.776)
Gender (ref: male)				
Female	0.999	(0.781—1.278)	0.867	(0.694—1.083)
Age (ref: 18–25 years)				
26–35	0.713	(0.436—1.166)	1.024	(0.693—1.512)
36–45	1.680	(0.979—2.882)	0.953	(0.607—1.495)
> = 46	2.012*	(1.133—3.574)	0.946	(0.588—1.521)
Residency (ref: local residents)				
Migrants	1.479*	(1.040—2.104)	1.107	(0.818—1.499)
Marital status (ref: single)				
Married	0.761	(0.500—1.159)	0.897	(0.624—1.290)
Divorced/widow	1.092	(0.479—2.490)	0.567	(0.262—1.224)
Educational attainment (ref: middle school or lower)				
High school or technical secondary school	1.085	(0.652—1.807)	1.206	(0.873—1.667)
Junior college	1.996**	(1.236—3.225)	1.023	(0.734—1.425)
Bachelor's degree or higher	2.473**	(1.494—4.094)	1.024	(0.716—1.465)
Occupation (ref: government agency)				
Service industry	0.285**	(0.214—0.381)	1.010	(0.756—1.348)
Manufacturing industry or agriculture	0.104**	(0.063—0.171)	1.008	(0.718—1.416)
Others	0.119**	(0.081—0.175)	0.846	(0.626—1.145)
Annual individual income (ref: < 20 k)				
20-50 k	1.487	(0.909—2.432)	0.939	(0.650—1.358)
50-100 k	1.061	(0.672—1.677)	0.905	(0.644—1.273)
100-200 k	0.719	(0.445—1.163)	0.955	(0.661—1.379)
> 200 k	0.747	(0.441—1.265)	1.106	(0.724—1.689)
Self-reported health status (ref: good)				
Poor	0.540**	(0.352—0.829)	0.757	(0.543—1.056)
Awareness of COVID-19 (ref: low)				
High	1.683	(0.991—2.859)	3.391**	(2.285—5.032)
Perceived susceptibility of COVID-19 (ref: low)				
High	3.457**	(2.298—5.199)	1.950*	(1.119—3.398)
Perceived severity of COVID-19 (ref: mild)				
Severe or moderate	0.844	(0.629—1.133)	1.213	(0.933—1.578)

^*^p < 0.05. **p < 0.01

*aOR* adjusted odds ratio

Only 1714 participants who haven't received or scheduled a COVID-19 vaccine were asked their willingness. Among them, three participants didn't report their willingness, so the sample size for the vaccination willingness question was limited to 1711

## Discussion

By early 2021, about half (52.45%) of adults aged 18 years and older had received or scheduled a COVID-19 test at least once, and the majority (95.63%) of total respondents reported being willing to receive a test in the future; about two-thirds (63.28%) of respondents had received/scheduled a vaccination or were willing to be vaccinated against COVID-19 in the future. Higher willingness and uptake of COVID-19 testing were associated with more secure occupations, while associations with other socio-demographic characteristics failed to reach statistical significance. Being of older age and migrants, having higher educational attainment and working in a secure job were associated with higher uptake of COVID-19 vaccinations, while willingness to receive a vaccination was consistent across the various socio-demographic characteristics assessed. High awareness of and perceived susceptibility to COVID-19 were associated with higher willingness and uptake of COVID-19 testing and vaccination.

Adults in China expressed almost universal willingness to receive COVID-19 testing (95.63%); this rate is higher than populations observed in some other countries, such as Ethiopian and Japan [60, 61]. Regarding the uptake of COVID-19 testing, although some areas had not reported any confirmed cases, over half of respondents reported having received or scheduled a test. Except for occupation, no socio-demographic characteristic was associated with the willingness and uptake of COVID-19 testing, which is consistent with previous studies [53, 62]. This indicated the equal willingness and access to COVID-19 testing in China, which may lie in the fact that the Chinese central government and some local governments require healthcare providers to supply the community-wide mass COVID-19 testing without cost-sharing and the testing policies for travel [63]. As a result, we found that the majority of tests were performed due to community-wide mass testing and mandatory testing policies for travel. This finding provides some evidence to support the rollout of mass-testing in urban regions of China. The different uptake rates by occupation may result from the requirements of the employers and government, which may alleviate the anxiety to go back to work in person and be assist in preventing and controlling confirmed cases as well. During the current phase of the pandemic with only sporadic cases, mass testing policies appear to be supported by the public, and continuous implementing these policies could help identify, trace and mitigate confirmed cases even during the low-risk period of the COVID-19 pandemic in China.

As China entered the low-risk period of the COVID-19 pandemic, willingness of the COVID-19 vaccination appears to become relatively low, with only 63.28% of total respondents having received/scheduled or being willing to receive a vaccination, which could pose a challenge to the achievement of herd immunity. The willingness of COVID-19 vaccination in our study is lower than high willingness (around 90%) found in mid-2020 when the peak of the pandemic had just passed in China [30, 46, 64, 65]. This reduced willingness to vaccinate could be due to the successful prevention and control of the pandemic and low risks of COVID-19 infections in China [4]. Concerns regarding the efficacy and safety of the COVID-19 vaccination may be another reason for individuals not being willing to receive a vaccination [46, 65, 66]. Thus, the importance, efficacy, and safety of COVID-19 vaccination should be emphasized more to maintain high willingness of vaccination especially when the pandemic evolved into the low-risk period. In fact, vaccination willingness had been observed to rebound with outbreaks in several cities in 2021 [67]. For example, following the outbreak in Guangzhou city in May 2021, uptake of both doses of the COVID-19 vaccination has quickly reached 70% within a month in Guangzhou [68] and the nearby city, Shenzhen [69].

Among adults who had not been vaccinated or scheduled a vaccination, socio-demographic characteristics were not associated with willingness to receive a vaccination, indicating the equitable vaccination willingness in China [21, 40]. However, the uptake of vaccinations differed by age, residence, educational attainment, and occupation. These disparities are aligned with previous studies in other countries that reported a higher vaccination uptake among older adults and those with higher socioeconomic status [28, 29, 31, 36, 70–72]. In China, the COVID-19 vaccination is free to the public, [73] and to improve the access to the vaccination, COVID-19 vaccination units are temporarily set up within the community providing walk-up COVID-19 vaccination [74]. Older adults and migrants face more risks of infecting COVID-19, and are the priority groups of vaccination. Although with equitable willingness, population with a higher level of educational attainment and working in a secure job face more policy requirements and less barriers to be vaccinated than their counterparts. Most of them work at government agency and formal employment, which usually require the employees to be fully vaccinated before returning to the office in person. This policy requirements may contribute to the higher uptake of vaccination among those with a higher level of educational attainment and working in a secure job.

In addition, higher awareness of and perceived susceptibility to COVID-19 were positively associated with the respondent’s willingness to receive and uptake of COVID-19 testing and vaccination, which concurs with data from previous studies reporting that perceiving a high risk of infections may increase willingness and uptake rates for both testing and vaccination [29, 33, 42–47, 53, 62, 75, 76].

This study is subject to several limitations. First, this study conducted an online survey, which may have resulted in the study groups being more homogenous with respect to certain socio-demographics. Second, this cross-sectional study just showed associations, instead of causal associations, and did not compare the current rates of willingness to receive and uptake of COVID-19 testing and vaccination with the rates from the active period of the pandemic, and therefore cannot capture trends or the changes in these proportions over time. Last, this study was conducted at the early stage of COVID-19 vaccination rollout. Thus, further research is warranted to assess the public’s uptake and willingness to receive COVID-19 testing and vaccination in different phases of the pandemic.

This study initially estimated the willingness and uptake of COVID-19 testing and investigated the risk factors associated with the testing, which may assist in identifying the tailored population to be affected by health education interventions. This study also measured the willingness and uptake of COVID-19 vaccination when the COVID-19 pandemic evolved into the low-risk period, helping identify how the willingness changes over time and supporting in the evaluation of relevant policies on increasing the use of COVID-19 vaccination.

## Conclusion

By early 2021, Chinese adults expressed almost universal willingness of COVID-19 testing, and over half of adults have been tested, which may be associated with a community-wide mass testing and traveler testing policies in China. The willingness and uptake of COVID-19 vaccination were relatively low at the low-risk period of the COVID-19 pandemic, and the uptake was independent from socio-demographic characteristics at most cases. Maintaining public willingness, enhancing public trust, and eliminating disparities in the uptake of COVID-19 vaccination may assist in improving the willingness and update of COVID-19 vaccination.

## Data Availability

We confirm that we had full access to all the data in the study and take responsibility for the integrity of the data and the accuracy of the data analysis as well as the decision to submit for publication. The datasets used and/or analysed during the current study available from the corresponding author on reasonable request.
